# Molecular Targeted Therapies in Glioblastoma Multiforme: A Systematic Overview of Global Trends and Findings

**DOI:** 10.3390/brainsci13111602

**Published:** 2023-11-17

**Authors:** Emir Begagić, Ragib Pugonja, Hakija Bečulić, Amila Čeliković, Lejla Tandir Lihić, Samra Kadić Vukas, Lejla Čejvan, Rasim Skomorac, Edin Selimović, Belma Jaganjac, Fatima Juković-Bihorac, Aldin Jusić, Mirza Pojskić

**Affiliations:** 1 Department of General Medicine, School of Medicine, Unversity of Zenica, Travnička 1, 72000 Zenica, Bosnia and Herzegovina; begagicem@gmail.com (E.B.);; 2 Department of Anatomy, School of Medicine, University of Zenica, Travnička 1, 72000 Zenica, Bosnia and Herzegovina; rpugonja@gmail.com; 3 Department of General Medicine, Primary Health Care Center, Nikole Šubića Zrinjskog bb., 72260 Busovača, Bosnia and Herzegovina; 4 Department of Neurosurgery, Cantonal Hospital Zenica, Crkvice 76, 72000 Zenica, Bosnia and Herzegovina; 5 Department of Neurology, Cantonal Hospital Zenica, Crkvice 76, 72000 Zenica, Bosnia and Herzegovina; 6 Department of Surgery, School of Medicine, University of Zenica, Travnička 1, 72000 Zenica, Bosnia and Herzegovina; edin.selimovic@unze.ba; 7 Department of Histology, School of Medicine, University of Zenica, Travnička 1, 72000 Zenica, Bosnia and Herzegovina; belma.jaganjac@gmail.com (B.J.);; 8 Department of Pathology, School of Medicine, University of Zenica, Travnička 1, 72000 Zenica, Bosnia and Herzegovina; 9 Department of Pathology, Cantonal Hospital Zenica, Crkvice 76, 72000 Zenica, Bosnia and Herzegovina; 10 Department of Neurosurgery, University Hospital Marburg, Baldingerstr., 35033 Marburg, Germany

**Keywords:** target therapy, glioblastoma, central nervous system, molecular biology

## Abstract

This systematic review assesses current molecular targeted therapies for glioblastoma multiforme (GBM), a challenging condition with limited treatment options. Using PRISMA methodology, 166 eligible studies, involving 2526 patients (61.49% male, 38.51% female, with a male-to-female ratio of 1.59/1), were analyzed. In laboratory studies, 52.52% primarily used human glioblastoma cell cultures (HCC), and 43.17% employed animal samples (mainly mice). Clinical participants ranged from 18 to 100 years, with 60.2% using combined therapies and 39.8% monotherapies. Mechanistic categories included Protein Kinase Phosphorylation (41.6%), Cell Cycle-Related Mechanisms (18.1%), Microenvironmental Targets (19.9%), Immunological Targets (4.2%), and Other Mechanisms (16.3%). Key molecular targets included Epidermal Growth Factor Receptor (EGFR) (10.8%), Mammalian Target of Rapamycin (mTOR) (7.2%), Vascular Endothelial Growth Factor (VEGF) (6.6%), and Mitogen-Activated Protein Kinase (MEK) (5.4%). This review provides a comprehensive assessment of molecular therapies for GBM, highlighting their varied efficacy in clinical and laboratory settings, ultimately impacting overall and progression-free survival in GBM management.

## 1. Introduction

Glioblastoma multiforme (GBM) is the most common primary brain tumor in adults, representing 45.2% of malignant brain and CNS tumors [1,2,3,4]. It is classified as a grade IV diffuse astrocytic glioma by the World Health Organization (WHO) due to its invasive growth and specific histopathological and immunohistochemical features [5]. Molecular targeted therapies have emerged as a promising avenue for addressing GBM’s complexity and limited treatment options [6,7,8,9,10,11]. Frequent genetic alterations, such as p53 mutations, EGFR amplification, CDKN2a deletion, and PTEN mutations, offer potential therapeutic targets [11,12,13,14,15,16,17,18,19,20,21]. Current treatments, including surgery, radiation, and chemotherapy, yield a median survival of only 15 months for GBM patients, with frequent aggressive recurrences [12]. Patients also contend with significant psychological challenges that impact their quality of life [14].

This systematic review is driven by the critical need to consolidate and analyze key advancements in the field of molecular targeted therapies for GBM. Despite ongoing efforts, the complex nature of GBM and limited treatment options emphasize the significance of evaluating current research directions. Our primary goal is to offer crucial insights to the scientific community and healthcare professionals, contributing to the quest for more effective molecular interventions and improved outcomes for GBM patients.

## 2. Materials and Methods

A comprehensive systematic analysis was conducted to assess the present status of molecular targeted treatments for gliomas, aimed at providing valuable insights for scientific advancement and steering progress in this research domain. The methodology adhered to the established PRISMA (Preferred Reporting Items for Systematic Reviews and Meta-Analyses) guidelines [22]. This systematic review was registered in the Open Science Framework (OSF) registry under the identifier OSF-REGISTRATIONS-UBGYC-V1.

### 2.1. Search Strategy

In March 2023, a literature search of English-text articles was conducted using PubMed and Web of Science. Categories of concepts related to molecular targeted therapy were explored, focusing on Glioblastoma multiforme (GBM) and excluding other specific types. The search query used was (Glioblastoma multiforme OR GBM) AND (Molecular targeted therapy OR Protein Kinase Inhibitors OR Immunotherapy OR Apoptosis) from 2000 to 2022. Details about the search methodology are provided in Appendix A.

### 2.2. Inclusion and Exclusion Criteria

The screening and analysis process involved multiple authors to ensure rigor and accuracy. Initially, article titles and abstracts were assessed by four authors. Subsequently, the remaining articles underwent meticulous examination by a panel of five authors. To ensure the highest level of precision, the screening process was carried out in multiple stages. Initially, two authors evaluated article titles and abstracts for relevance, with a focus on removing any duplicate entries. Following this initial phase, the remaining articles underwent comprehensive full-text scrutiny by three authors.

**Figure 1:**
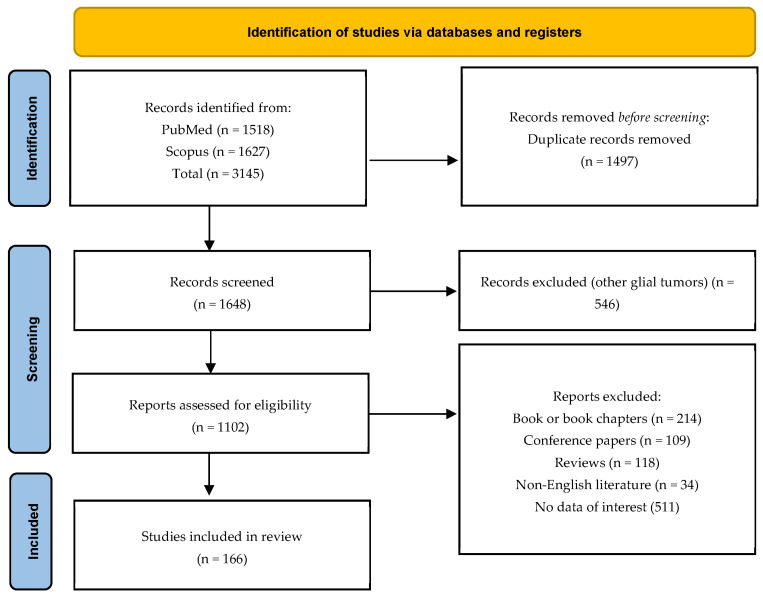
PRISMA flowchart.

The inclusion criteria were rigorously adhered to, encompassing studies that met the following criteria: (1) clinical studies, (2) laboratory studies, (3) molecular targeted therapies designed specifically for GBM, (4) studies involving adult participants, and (5) studies from 2000 to 2022. Exclusion criteria were applied as follows: (1) book or book chapters, (2) conference papers, (3) narrative and systematic reviews, (4) non-English literature, (5) studies lacking data of interest (including those related to other glial tumors or studies without predefined data for extraction), and (6) studies involving pediatric populations (Figure 1).

### 2.3. Data Extraction and Processing

In the systematic review, data extraction encompassed several key elements. These comprised the primary author’s name, year of publication, geographical location, study design, number of subjects (if applicable), molecular target, associated molecular pathway, as well as the approach used and principal discoveries. For the purposes of this study, categorization was performed based on the molecular mechanisms targeted by therapy. The classification is further detailed in Table 1.

### 2.4. Statistical Analysis and Graphical Elements

The statistical analysis was conducted using IBM SPSS Statistics (Version 27.0., International Business Machines Corporation, Armonk, NY, USA). The analysis encompassed the processing of categorical variables, with their presentation in the form of frequencies and percentages. Graphical representations were generated for research purposes in non-commercial platforms (Google Sheets and Google Drawings). Elements utilized for depicting molecular pathways were sourced from the non-commercial database, Servier Medical Art (SMART, Manila, Philippines).

## 3. Results

### 3.1. Global Research Trends

**Figure 2:**
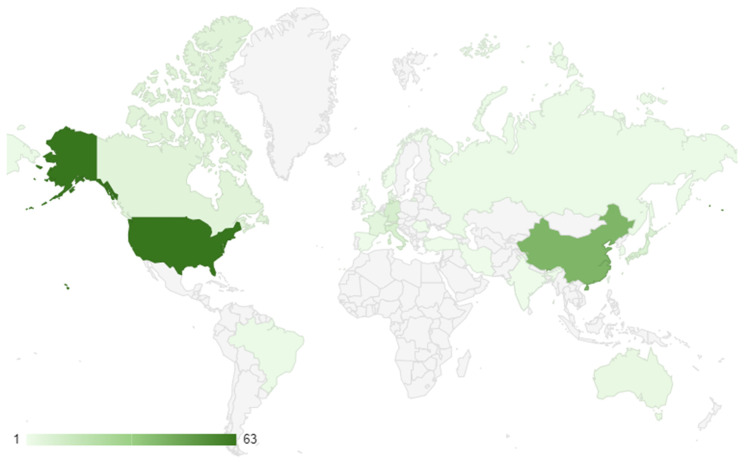
Geographical distribution of research conduction.

A total of 166 studies met the eligibility criteria for the systematic review [23,24,25,26,27,28,29,30,31,32,33,34,35,36,37,38,39,40,41,42,43,44,45,46,47,48,49,50,51,52,53,54,55,56,57,58,59,60,61,62,63,64,65,66,67,68,69,70,71,72,73,74,75,76,77,78,79,80,81,82,83,84,85,86,87,88,89,90,91,92,93,94,95,96,97,98,99,100,101,102,103,104,105,106,107,108,109,110,111,112,113,114,115,116,117,118,119,120,121,122,123,124,125,126,127,128,129,130,131,132,133,134,135,136,137,138,139,140,141,142,143,144,145,146,147,148,149,150,151,152,153,154,155,156,157,158,159,160,161,162,163,164,165,166,167,168,169,170,171,172,173,174,175,176,177,178,179,180,181,182]. The research trends showed that the majority of the studies were conducted in the USA, with 63 studies (38.0%) (Figure 2). China had the second-highest number of studies, with 41 (24.7%), followed by Germany with 10 (6.0%), Italy with 9 (5.4%), and Japan with 8 (4.8%). Other countries with a significant number of studies include France (5; 3.0%), Canada (6; 3.6%), and Australia (3; 1.8%). The remaining countries had one or two studies each, with India, Iran, Korea, Luxembourg, Norway, Romania, Russia, Spain, Switzerland, Taiwan, Turkey, and the United Kingdom each having one study.

**Figure 3:**
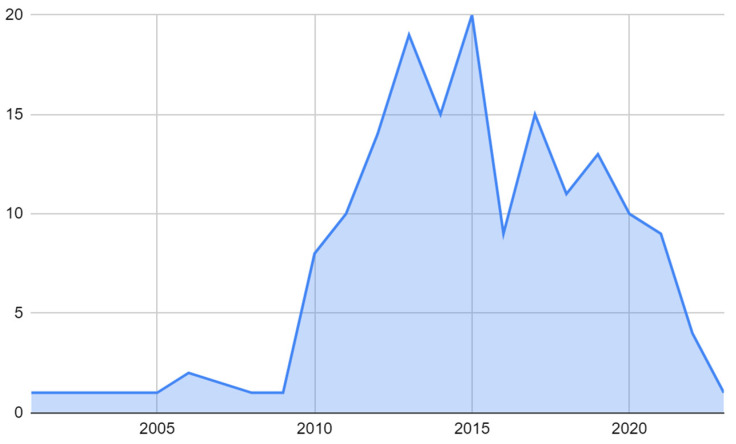
Temporal distribution of research of molecular target therapy of GBMs.

The studies included in the review spanned from 2001 to 2022, with the majority of the studies conducted between 2013 and 2015, accounting for 11.4% and 12.0% of the total studies, respectively. The next highest number of studies took place in 2012, with 8.4% of the total studies. The years with the least number of studies were 2001, 2003, 2004, 2005, 2008, 2009, and 2017, each with only one study (Figure 3). 

### 3.2. Study Design, Type of Target Therapy, and Molecular Mechanisms

The comprehensive systematic review incorporated a total of 27 studies (constituting 16.3% of the total) focused on clinical applications, and a substantial majority of 139 studies (making up 83.7%) were conducted within controlled laboratory environments. 

**Figure 4:**
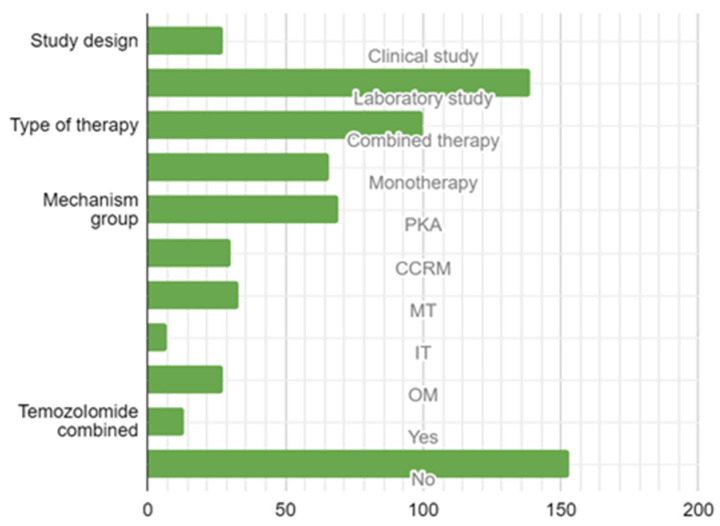
Study design, type of targeted therapy, mechanism, and combination with temozolomide. Legend: PKP—Protein Kinase Pathway Group; CCRM—Cell Cycle-Related Mechanisms; MT—Microenvironmental Mechanisms; IT—Immunomodulatory Targets; OT—Other Targets.

Within the domain of therapeutic modalities, a significant proportion of 100 studies (60.2%) embraced a multifaceted therapeutic approach, while a slightly smaller portion of 66 studies (39.8%) concentrated on mono-therapeutic strategies. In terms of mechanistic classification, 69 studies (41.6%) were categorized under the PKP mechanism, 30 studies (18.1%) were classified under CCRM, 33 studies (19.9%) were designated under Microenvironmental Targets (MT), 7 studies (4.2%) fell under IT, and 27 studies (16.3%) were attributed to OM (Figure 4).

The most frequently encountered molecular target was found to be the Epidermal Growth Factor Receptor (EGFR), accounting for a substantial 18 instances (10.8%). Following closely were the Mammalian Target of Rapamycin (mTOR) with 12 occurrences (7.2%), Vascular Endothelial Growth Factor (VEGF) with 11 instances (6.6%), and Mitogen-Activated Protein Kinase (MEK) with 9 cases (5.4%). Phosphoinositide 3-Kinase (PI3K) and B-Raf Proto-Oncogene (BRAF) exhibited an equal number of occurrences, each accounting for 8 cases (or 4.8%), while they were attributed to 5 cases (3.0%), respectively.

**Figure 5:**
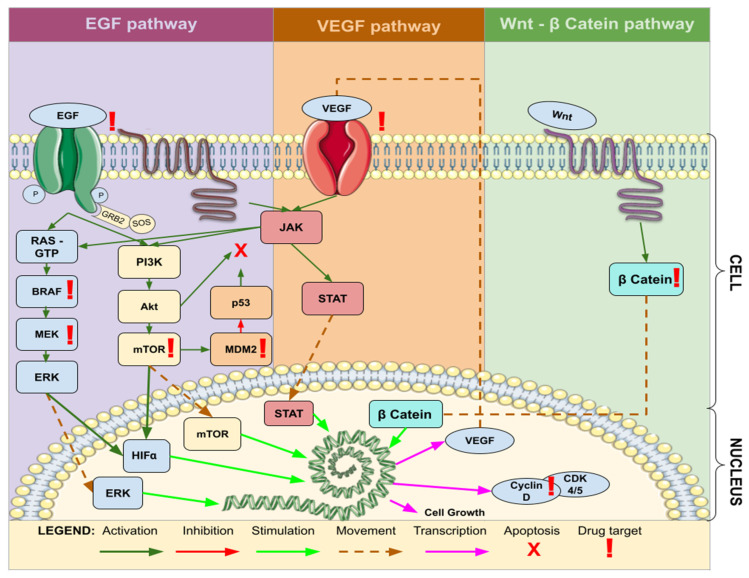
Common molecular pathways associated with target therapy of GBM. Legend: EGF—Epidermal Growth Factor; VEGF—Vascular Endothelial Growth Factor; JAK—Janus Kinase; STAT—Signal Transducer and Activator of Transcription; Wnt—Wingless-Related Integration Site; Cyclin—Regulatory proteins involved in cell cycle progression; β Catenin—Beta-Catenin; RAS—Rat Sarcoma; GTP—Guanosine Triphosphate; BRAF—B-Raf Proto-Oncogene; MEK—Mitogen-Activated Protein Kinase Kinase; ERK—Extracellular Signal-Regulated Kinase; PI3K—Phosphatidylinositol 3-Kinase; Akt—Protein Kinase B; mTOR—Mammalian Target of Rapamycin; HIFa—Hypoxia-Inducible Factor alpha; CDK—Cyclin-Dependent Kinase; MDM2—Mouse Double Minute 2 Homolog.

VEGF, known as Vascular Endothelial Growth Factor, induces an augmentation in the vascularization of GBM. Consequently, it is categorized within the Endothelial Targets (ET) group, despite subsequently activating the Protein Kinase Phosphorylation (PKP) mechanism, akin to EGFR. With respect to Immunological Targets (IT), it encompasses molecular targets such as Extracellular Matrix Metalloproteinase Inducer (EMMPRIN), Autotaxin (ATX), and Lysophosphatidic Acid (LPA), which are associated with the ATX–LPA pathway. This pathway eventually activates Beta Catenin, emerging as a significant avenue of interest in the context of targeted therapy for GBM (Figure 5).

### 3.3. Findings from Clinical Studies

The total number of patients involved in 27 clinical studies is 2526, with three studies not reporting gender distribution numbers (Table 2). Among the known gender distribution data for 1244 patients, 764 (61.49%) were male and 480 (38.51%) were female, resulting in a male-to-female ratio of 1.59/1. The lowest recorded median age was 49 years, while the highest was 90 years. Upon examining the interquartile ranges, it is observed that the youngest participant in these studies was 18 years old, while the oldest was 100 years old.

In the context of GBM target therapy treatment, various therapeutic approaches and drug regimens have been explored, each yielding distinct success rates and outcomes. Notably, Imatinib exhibited no significant effect on GBM, with a median progression-free survival (mPFS) of 2.8 months (and control: 2.1 months), showing no statistical significance between the investigated and control groups [145]. In contrast, Nimotuzumab combined with temozolomide and radiation therapy resulted in similar survival times, boasting a median overall survival (mOS) of 15.9 months and a median progression-free survival (mPFS) of 10 months [165]. In the study by Desjardins et al. [54], the combination of bevacizumab with temozolomide showed activity and tolerance, with a median progression-free survival (mPFS) of 15.8 weeks. In the research conducted by Brown et al. [37], the combination of Bevacizumab with Cediranib and Gefitinib demonstrated improved progression-free survival, resulting in a progression-free survival (PFS) of 3.6 months. Additionally, Badruddoja et al. [29] found that bevacizumab, when combined with temozolomide, served as a salvage regimen for recurrent GBM, with an overall response rate from diagnosis of 51 weeks, a PFS-6 of 52%, and a median time to tumor progression of 5.5 months. Regorafenib demonstrated a survival benefit in recurrent GBM, with a survival of 24.8 months [109], while Pembrolizumab, with or without bevacizumab, proved ineffective in therapy, resulting in a progression-free survival rate of 26.0% and an overall survival of 8.8 months with bevacizumab, and a progression-free survival rate of 6.7% and an mOS of 10.3 months without bevacizumab [124]. These findings highlight the diverse landscape of therapeutic strategies and their associated outcomes in the management of GBM.

### 3.4. Findings from Laboratory Studies

Out of a total of 139 laboratory studies, the most common research samples were human GBM cell lines, specifically human cell cultures (HCC), accounting for 73 studies (52.52%). Subsequently, there were 60 studies (43.17%) that utilized animal samples, and 6 studies (4.32%) employed a combination of sample sources. 

In animal studies, mice were predominantly used as the sample (52 studies), representing 37.41%.

Various drugs and treatment combinations demonstrated significant anti-glioma effects, including the inhibition of glioma proliferation, reduced invasion, enhanced apoptosis, and extended survival. Particular highlights include the effectiveness of O-acetyl GD2 ganglioside, Amb4269951, rSLURP-1, ILK inhibition, AAL881, and the combined mTOR1 and MEK1/2 inhibition in CDK4-dysregulated tumors. Moreover, the exploration of various molecular targets, such as EGFR, EGFRvIII, miRNAs, MET, and other signaling pathways, underscores the complex nature of glioma and the potential for targeted therapies.

#### 3.4.1. Overview of In Vitro Laboratory Studies

The total number of in vitro studies included in the systematic review amounted to 42, constituting 25.3% of the overall study count. The GBM cell lines most frequently encountered in these studies were the U87 cell line (comprising 17 studies, or 40.5%), which featured prominently across various investigations. Following this, the U251 cell line (noted in 11 studies, or 26.2%) and the T98G cell line (present in 10 studies, or 23.8%) were also commonly employed.

Regarding potential drugs for the treatment of GBM, numerous compounds exhibited promise within the in vitro research. Particularly, Sorafenib, functioning as a multi-kinase inhibitor, showcased robust anti-glioma activity in both in vitro settings, as emphasized in the study by Siegelin et al. [132]. Furthermore, the combination of Metformin and Sorafenib was identified as an effective treatment strategy for TMZ-resistant GBM cells, as demonstrated in the investigation conducted by Aldea et al. [24]. The research by Paternot et al. [128] underscored the potential of Rapamycin and PD184352 as a combined therapeutic approach, effectively inhibiting DNA synthesis and pRb phosphorylation, especially in CDK4-dysregulated tumors (Table 3).

#### 3.4.2. Overview of In Vivo Laboratory Studies

The systematic review encompassed a total of 62 in vivo studies, constituting 37.4% of the overall studies included in the analysis. Among these in vivo studies, the GBM cell line U87-MG was the most prominently observed (comprising 9.67% of the total), with GSC11 and U251-MG cell lines each being mentioned in two studies. Of these in vivo studies, the majority (87%) involved animal subjects, with a predominant focus on mouse samples (74.2%). Two studies (3.2%) reported human population involvement.

Regarding potential drugs for GBM treatment, the provided studies showcased several promising therapeutic approaches. For instance, AMB4269951, as elucidated in the investigation by Takano et al. [152], demonstrated remarkable anti-tumor effects against gliomas. Rslurp-1, as evidenced by the research conducted by Saito et al. [139], exhibited notable antitumor activity, resulting in increased survival rates. AA1881, explored in the study led by Sathorn-Sumetee et al. [143], targeted BRAF, CRAF, and VEGFR, yielding inhibition of glioma growth and an extension in median survival (Table 4). 

#### 3.4.3. Overview of Combined Laboratory Studies

Table 5 furnishes an overarching perspective on the amalgamation of in vivo and in vitro investigations pertaining to GBM, constituting a total of 32 combined studies (19.3%). One conspicuous facet of these studies is the breadth of molecular mechanisms and targets that they explore. For example, Kuan et al. [97] concentrate on receptor-based targeting strategies, with specific regard to TfR (transferrin receptor), while Guo et al. [71] delve into the realm of kinase inhibitors, particularly CDK 4/6 and PDGFRα. Moreover, various studies scrutinize molecular targets encompassing EZH2, FPR, JNK, and PI3K, thereby highlighting the intricate and multifaceted landscape of GBM.

These investigations also shed light on the efficacy of the therapies, with numerous studies presenting encouraging outcomes in terms of extended survival and tumor regression. For instance, Rslurp-1, Dasatinib, GNE-317, and dual mTOR1/2 inhibition yield augmented survival rates, signifying their potential utility in GBM treatment. Furthermore, the juxtaposition of therapies such as TRAIL and TMZ or PDK1 and CHK1 inhibitors reveals synergistic effects in the inhibition of tumor growth (Table 5).

## 4. Discussion

### 4.1. Global and Research Trends of GBMs

The global incidence of CNS tumors in 2019 was reported at 347,992 cases, indicating a substantial 94.35% increase from the period spanning 1990 to 2019 [183]. Notably, the incidence of brain tumors exhibited significant regional variation, with the highest rates observed in North America and the lowest in Africa. This trend was found to correlate with increasing Gross Domestic Product (GDP) per capita [184]. 

Examining the temporal distribution of studies in this systematic review, a notable proportion were conducted between 2013 and 2015, collectively accounting for 23.4% of the total studies. This surge in research activity post-2000s appears to be closely linked to the escalating incidence of GBM. Grech et al.’s [185] research unveiled a significant increase in GBM incidence from 2010, accompanied by a noteworthy increase in incidence risk ratio, measured at 1.16 per additional year. Projections further anticipate a 72% surge in incidence by 2050, compared to figures from 2010 [186].

Within this systematic review, clinical studies constituted 27 (16.3%) of the studies, while laboratory studies comprised the majority, accounting for 139 (83.7%). This distribution reflects the inherent challenges associated with limited patient cohorts and abbreviated survival durations. Initially perceived as predominant in developed nations, oncological diseases like GBM are now assuming the role of a significant economic and health burden in low- and middle-income countries (LMICs) [187]. The management of GBM in these settings is hindered by escalating financial constraints, a shortage of clinical trials, and restricted access to first-line therapeutic agents. The scarcity of healthcare professionals and the suboptimal quality of care further exacerbate the treatment gap for GBM in these regions [187]. Consequently, GBM imposes a substantial financial strain on the healthcare systems of impoverished nations [188,189,190,191,192,193,194,195]

### 4.2. Current State of Targeted Molecular Therapy in GBM Treatment

The prevailing standard of care for GBM involves the maximal surgical removal of the tumor, followed by localized chemotherapy utilizing TMZ, a second-generation imidazotetrazine known for its DNA-alkylating properties [196]. Its ability to penetrate the blood-brain barrier makes it particularly potent in treating brain tumors [197]. However, alongside its benefits, TMZ is associated with significant side effects such as myelotoxicity, ulcers, nausea, vomiting, fatigue, and harmful DNA damage. Moreover, resistance to this drug is commonplace in GBM patients [198]. To enhance the effectiveness of initial GBM treatment, it may be worthwhile to investigate a more potent combination regimen [199]. The presented findings in this review pertain to the use of therapeutic methods and chemotherapeutic agents in the treatment of GBM. These results reveal that a substantial majority of studies (60.2%) advocated for a comprehensive therapeutic approach, while a slightly smaller portion (39.8%) focused on single-strategy treatments. 

In terms of mechanistic categorization, 41.6% of studies fell into the PKP mechanism, 18.1% were classified as CCRM, 19.9% were designated as Microenvironmental Targets (MT), 4.2% were categorized as IT, and 16.3% were attributed to OM. Currently, the predominant chemotherapeutic compounds employed in the management of GBM are small molecules designed to intervene with specific aberrant signaling pathways within GBM cells, including receptor tyrosine kinase activity, the PI3K/AKT/mTOR cascade, the cellular response to DNA damage, TP53 function, and inhibitors of the cell cycle [200]. The disrupted regulation of numerous signaling pathways in GBM serves as the primary catalyst for the uncontrolled proliferation of both initial and recurring tumors. This underscores the critical importance of identifying the optimal combination of targeted therapeutics for GBM treatment. It is noteworthy that most GBMs do not exhibit a singularly aberrant pathway, rendering them less amenable to targeted therapeutic approaches. This is exemplified by the lack of success observed in late-stage clinical trials of various targeted agents for GBM [200]. The most recent molecular and genomic evidence highlights the presence of diverse genetic and molecular characteristics within and between tumors in GBM [200]. This leads to variations in the expression of therapeutic targets across different tumors and regions within a single tumor. This heterogeneity in GBM may elucidate the lack of success observed in targeted treatments aimed specifically at tumor biomarkers, including drugs like cetuximab, gefitinib, erlotinib (targeting EGFR), bevacizumab (targeting VEGF), and cilengitide (targeting integrin). It is recognized as the underlying cause of resistance to these therapies.

Temozolomide, akin to dacarbazine, is an imidazotetrazine derivative. It stands out as one of the rare drugs capable of exerting its effects within the central nervous system [201]. In the treatment of GBM, TMZ’s primary mechanism of action involves methylating the O6 positions of guanine. This modification hinders DNA replication during cellular proliferation and triggers programmed cell death, or apoptosis. Following its approval by the FDA in 2005 [202], TMZ, when administered alongside surgery and radiotherapy, has solidified its position as the established and pivotal standard of care for individuals with GBM. This marked a significant milestone, as it rose to prominence as the leading initial chemotherapeutic option for GBM treatment. Findings from this study revealed that TMZ was utilized in 28% of the studies as part of a treatment regimen in conjunction with other molecular targeted therapy drugs.

In contemporary practice, TMZ is administered alongside radiotherapy as the primary treatment for GBM and as a secondary option for other malignant gliomas in cases of relapse. However, the utilization of radiotherapy and chemotherapy comes with certain limitations, and the emergence of tumor drug resistance is a common outcome. Beyond the known factors contributing to TMZ resistance, such as uncontrolled signaling pathways, DNA repair mechanisms, the persistence of cancer stem cell (CSC) subpopulations, and the activation of self-defense mechanisms [203], it is worth delving into alternative approaches that may hold promise in addressing these challenges. Mesenchymal stem cells (MSCs) are gaining traction as a therapeutic avenue in the field of cancer immunotherapy [204]. The development of chemoresistance to TMZ may arise from genetic and epigenetic alterations induced by the drugs in cancerous cells. These changes encompass the induction and selection of genes that confer a survival advantage, or the preferential selection of pre-existing cell clones with resistance. Potential alterations encompass an upsurge in drug efflux facilitated by active membrane pumps, deactivation of intracellular drugs, heightened resilience to DNA damage, and modifications in genes linked to apoptosis. These adjustments hold substantial importance in extensively heterogeneous tumors such as GBM, as treatment interventions may inadvertently promote the survival of resistant cells, potentially culminating in tumor recurrence. Nevertheless, there is evidence suggesting that combining TMZ with other molecular targeted therapies has demonstrated an improved survival rate [199].

The acquired resistance pathways in GBM involve the Src tyrosine kinase pathway, which regulates actin dynamics and the invasion of malignant glial cells [205]. Src transmits signals from the extracellular matrix and interacts with various intracellular proteins, including integrins, Eph kinase, and growth factor receptors. GBM cells exhibit higher Src tyrosine kinase activity compared to normal brain cells [206,207]. In a study by Eom et al. [208], an Src tyrosine kinase inhibitor (PP2) was examined in combination with TMZ. The findings indicated that PP2 enhanced the in vitro radiosensitivity of malignant glioma cells and inhibited invasion and migration. However, in in vivo trials, the combination led to a statistically non-significant decrease in tumor volume. On a different note, other authors [79] discovered that suppressing Src family kinase signaling could impede bevacizumab-induced GBM cell invasion, suggesting a potential strategy for overcoming GBM treatment resistance. Certain studies propose that miRNA may serve as a predictive marker for the response to TMZ treatment in GBM patients. Certain researchers propose that when combined with specific drugs, standard-dose TMZ chemotherapy may lead to an improvement in progression-free survival. As an illustration, the administration of trans sodium crocetinate (TSC), a substance known for its ability to enhance oxygen delivery, alongside standard-dose TMZ and radiotherapy proved beneficial for 59 GBM patients in a phase I/II trial conducted by Gainer et al. [209]. The outcomes revealed that 36% of patients who received TSC were still alive after two years, in contrast to 27–30% of those who underwent the standard treatment. The authors proposed that administering TSC in conjunction with the standard treatment conferred an advantage in GBM therapy [209]. According to Vengoji et al. [158] the combination of afatinib with TMZ significantly postpones the progression of GBM. In a study by Sang-Soo et al. [93], a nanocomplex targeting MALAT1 was examined, and the authors suggested that silencing MALAT1, combined with TMZ, also provided a survival benefit. Other combinations involving TMZ, such as its combination with dual mTOR1/2 inhibition, have proven to be effective therapies for resistant GBM. Similarly, the combination of Metformin and sorafenib has yielded the same effect [210,211].

In this review, the most frequently targeted molecular entity was identified as the EGFR, accounting for a substantial proportion. Following closely were the mTOR, VEGF, and MEK. PI3K and BRAF exhibited an equal number of occurrences. EGFR amplification and mutation are the most prevailing genetic alterations, occurring in more than 50% of GBM [200,212]. EGFRvIII is the most common and highly oncogenic EGFR mutant in GBM, and imaging the status of EGFRvIII could be of great value in GBM treatment [212]. VEGF induces an augmentation in the vascularization of GBM and is categorized within the ET group, despite subsequently activating the PKP mechanism, akin to EGFR. VEGFR and PDGFR are overexpressed, amplified, and/or mutated in GBM, leading to uncontrolled cell proliferation, angiogenesis, migration, survival, and differentiation [213].

Different cell lines are widely used in scientific research as valuable tools for studying various biological processes and diseases, including GBM. In this systematic review, human GBM cell lines, specifically HCC, were the most commonly utilized research samples, comprising 52.52% of the included laboratory studies. The prominent use of cell lines in GBM research highlights their importance in providing a controlled and reproducible model system for investigating the molecular mechanisms underlying GBM development and testing potential therapeutic interventions. These cell lines, such as U87, U251, and T98G, have been extensively employed in numerous investigations, demonstrating their relevance and utility in advancing our understanding of GBM biology [63,69]. In vitro studies using GBM cell lines have contributed significantly to the identification and evaluation of potential drugs for GBM treatment. Within the systematic review, 25.3% of the included studies focused on in vitro research. Notably, the U87 cell line emerged as the most frequently encountered cell line in these studies, appearing in 40.5% of the investigations. This consistent utilization of the U87 cell line underscores its importance as a representative model for studying GBM in vitro [179].

### 4.3. Effectiveness of Targeted Therapy in GBM Treatment

Several drugs have shown promise in the context of GBM target therapy treatment, as indicated by various outcomes, including survival time, mPFS, PFS-6, and OS data from Table 2. For instance, AZD1775 demonstrated therapeutic concentrations and good tolerability [141]. Alectinib, Palbociclib, Temsirolimus, Idasanutlin, and Vismodegib were evaluated in the NCT Neuro Master Match trial, which utilizes GBM molecular signatures for treatment [169]. However, Imatinib did not show a significant effect on GBM, with an mPFS of 2.8 months in Arm A and 2.1 months in Arm B, along with corresponding mOS values of 5.0 and 6.5 months [145]. Nimotuzumab, when combined with temozolomide and radiation therapy, exhibited promising results, with an mOS of 15.9 months and an mPFS of 10 months [165]. Bevacizumab, used in various regimens, demonstrated diverse outcomes, from activity and tolerance [29,37,54] to serving as a salvage regimen for recurrent GBM [29]. Regorafenib presented a significant survival benefit in recurrent GBM, with an mOS of 24.8 months [109]. Conversely, pembrolizumab, with or without bevacizumab, did not prove effective, resulting in a PFS-6 of 26.0% and an mOS of 8.8 months with bevacizumab, and a PFS-6 of 6.7% and an mOS of 10.3 months without bevacizumab [124]. These findings not only highlight the potential of various therapies but also emphasize the importance of assessing survival times and progression-free intervals in evaluating treatment efficacy for GBM patients.

### 4.4. Promising Targeted Therapies for GBM Treatment

Various targeted therapies demonstrate promising GBM treatment potential. The Anti-GD2 antibody [36] specifically targets O-acetyl GD2 ganglioside, effectively preventing glioma proliferation. AMB4269951 [152] shows antitumor effects by targeting CTL1 and significantly improving mouse survival. rSLURP-1 [139] effectively inhibits GBM growth by targeting α7 nAChR. QLT0276 in DMSO [95] inhibits integrin-linked kinase (ILK), leading to decreased glioma cell invasiveness and down-regulated proliferation and invasion. AA1881 [143] targets BRAF, CRAF, and VEGFR, significantly increasing mouse survival. EF2-siRNA [175], targeting EF2-kinase, demonstrates increased survival in rats and inhibits cell migration. Furthermore, boronated EGFR MAB + Cetuximab [176] significantly enhances survival by targeting EGFR and EGFRvIII tumors. The combination of Rapamycin + PD184352 [128] offers promise in CDK4-dysregulated tumors by providing complete inhibition of DNA synthesis and pRb phosphorylation. Tamoxifen [61] induces apoptosis and presents potential therapeutic targets for GBM. PX-866 [96] inhibits PI3K/Akt and increases survival in mice. NVP-AEW541 + Dasatinib [151] through dual IGF1R and Src inhibition increases apoptosis in glioma cells. Sorafenib [132] exhibits potent in vivo and in vitro anti-GBM activity. Plumbagin [120] effectively inhibits glioma proliferation and induces apoptosis, especially when combined with radiation. T7-modified liposomes [97] effectively penetrate the blood-brain barrier (BBB). The combination of SB203580 + Rapamycin [51] significantly inhibits tumor growth by targeting SAPK2/p38 and mTORC1. Anti-bFGF siRNA [106] holds potential for glioma treatment by inducing apoptosis. Lenvatinib + Crenolanib + Abemaciclib + Palbociclib [71], targeting PDGFRα and CDK4/6 signaling, offers a potential GBM treatment. DMC nanoparticle-mediated EZH2-siRNA [161] decreases tumor size. Targeting ID2 with anti-ID2 siRNA [180] increases sensitivity and decreases glioma apoptosis. Finally, F2 procyanidins [146] downregulate FPR and exert cytotoxic effects in mouse models.

### 4.5. Advantages and Disadvantages in Molecular Targeted Therapy of GBM

Precision-targeted therapies are engineered to selectively target cancer cells, potentially mitigating the adverse effects of treatment [214]. This focused approach enhances therapeutic efficacy while minimizing collateral damage to healthy tissues. Furthermore, targeted therapies can synergize with complementary treatments like chemotherapy and radiation therapy, yielding improved outcomes for patients [215]. By tailoring these therapies to the specific genetic profile of the tumor, treatment effectiveness is optimized. Additionally, precise administration through controlled targeting enhances drug delivery to the tumor site, augmenting treatment efficacy while reducing systemic toxicity [216]. Also, by accumulating comprehensive data from large-scale studies on molecular targets, researchers can harness the power of artificial intelligence to develop predictive algorithms for patient outcomes and prognosis. This emerging field holds immense promise and aligns with the ongoing advancements in neurosurgery and medical technology [217].

While targeted therapies demonstrate remarkable efficacy against specific molecular targets, the emergence of resistance in tumors over time poses a significant challenge. These therapies may not be universally effective across all subtypes of GBM due to the tumor’s intrinsic heterogeneity, making the identification of reliable targets a complex endeavor [216,218]. Moreover, the cost associated with targeted therapies, coupled with potential insurance coverage limitations, may restrict patient access to these advanced treatments, especially in lower-middle-income countries. It is essential to note that, like many treatments, targeted therapies can also induce side effects, such as skin rash, diarrhea, and fatigue, which may impact the overall quality of life for patients undergoing treatment.

### 4.6. Limitations of the Study

The limitations of this systematic review primarily revolve around its inclusion criteria, which restricted the analysis to studies published in English, potentially excluding relevant research in other languages. Additionally, the presence of heterogeneity among the sampled studies, such as variations in patient populations, treatment approaches, and study designs, may introduce some degree of bias and make it challenging to draw uniform conclusions.

## 5. Conclusions

In conclusion, this systematic review provides insights into the global and research trends of GBM and the current state of targeted molecular therapy in GBM treatment. The increasing incidence of GBM, particularly in developed regions, presents a substantial healthcare and economic burden. The distribution of clinical and laboratory studies in this review reflects the challenges associated with limited patient cohorts and abbreviated survival durations, which are particularly pronounced in low- and middle-income countries. The standard of care for GBM primarily involves maximal surgical removal of the tumor and the use of TMZ. However, resistance to TMZ is common, and exploring more potent combination regimens is crucial for enhancing GBM treatment. The findings reveal that most studies advocate for a comprehensive therapeutic approach, and the mechanistic categorization shows the importance of targeting multiple pathways. The effectiveness of targeted therapy in GBM treatment varies, and promising therapies target various molecular entities. Precision-targeted therapies offer advantages in terms of efficacy and reduced collateral damage, but resistance, tumor heterogeneity, cost, and potential side effects remain significant challenges.

## Figures and Tables

**Table 1:** Categorization based on target therapy/pathways.

Group	Abbreviation	Explanation
Protein kinase pathway group	PKP	Mechanisms related to protein kinases.
Cell cycle-related mechanisms	CCRM	Mechanisms associated with cell cycle, apoptosis, and transcription pathways.
Microenvironmental mechanisms	MT	Mechanisms in the tumor’s surrounding environment, including angiogenesis, cell–cell adhesion, and iron/cation regulation.
Immunomodulatory targets	IT	Targets that modulate the immune response.
Other targets	OT	Targets not falling into the previous categories.

**Table 2:** Overview of clinical studies.

Reference	Year	N	Male/Female Ratio	Years (Median and IQR)	Study Design	Molecular Mechanism	Molecular Target	Therapy	Success Rate/Outcome	Main Findings
Sanai et al. [141]	2018	20	12/8	59 (28–81)	NRCT	PKP	Wee1K	AZD1775	N/A	AZD1775 reaches therapeutic concentrations in GBM, well tolerated.
Wick et al. [169]	2019	450	N/A	N/A	NRCT (II)	PKP	ALK CDK4/6 mTOR MDM2 SHH	Alectinib Palbociclib Temsirolimus Idasanutlin Vismodegib	N/A	NCT Neuro Master Match (N2M2) trial uses GBM molecular signatures for treatment.
Sauter et al. [145]	2022	51	36/15	Primary: 63 (35–78); Recurrent: 52.5 (29–70)	NRCT (II)	OM	CSF1R, ABL, cKIT, PDGFR	Imatinib	mPFS 2.8 m in arm A and 2.1 m in arm B. mOS was 5.0 (0.8–30) m in arm A and 6.5 m in arm B.	Imatinib shows no significant effect on GBM.
Wang et al. [165]	2014	92	21/5	50 (18–76)	Pilot clinical study	MT	EGFR	Nimotuzumab + TMZ + RT	mOS 15.9 m; mPFS 10 m.	Nimotuzumab, TMZ, and RT offer similar survival times.
Hasselbalch et al. [74]	2010	37	21/16	57.9 (23.8–70.3)	Prospective study	MT	EGFR, VEGF, topoisomerase I	Cetuximab + bevacizumab + irinotecan	mPFS in CBI (*n* = 37) corresponded to 17 w.	No biomarkers identify bevacizumab benefits.
Mason et al. [116]	2012	32	22/10	53 (43–71)	RCT (I)	PKP	mTOR1	Everolimus + TMZ	N/A	Everolimus (5 days) + TMZ is an appropriate phase II dose.
Chinnaiyan et al. [48]	2013	25	14/11	57 (31–73)	RCT (I)	PKP	mTOR	Everolimus + TMZ + RT	N/A	Everolimus (10 mg) + RT/TMZ: tolerable, acceptable toxicity.
Lassen et al. [99]	2015	22	32/12	60 (37–72)	RCT (I)	MT	Placental growth factor (PlGF) + VEGF	RO5323441 + Bevacizumab	mPFS 3.5 m, mOS 8.5 m.	RO5323441 plus bevacizumab toxicity is manageable.
Desjardins et al. [54]	2012	32	19/13	56 (25–80)	RCT (II)	MT	VEGF	Bevacizumab	MPFS was 15.8 w.	Combined temozolomide and bevacizumab show activity and tolerance.
Vredenburgh et al. [160]	2012	125	74/51	56.2 (19–80)	RCT (II)	MT	VEGF	Bevacizumab + RT + TMZ	Medaian PFS was 13.8 m.	Bevacizumab addition to TMZ and RT has minimal toxicity.
Zustovich et al. [182]	2013	43	18/25	60 (36.1–77.0)	RCT (II)	PKP	Multitarget kinase	Sorafenib	Median time-to-progression was 3.2 m.	Sorafenib + TMZ is safe with activity in relapsed GBM.
Clarke et al. [50]	2014	59	N/A	90 (60–100)	RCT (II)	MT	VEGF + tyrosine kinase	Bevacizumab + Erlotinib	OS: 19.8 m, PFS: 13.5 m.	Bevacizumab/erlotinib/TMZ/radiotherapy improves progression-free survival.
Brown et al. [37]	2016	38	27/11	57.0 (30–71)	RCT (II)	MT	VEGFR + EGFR	Cediranib + Gefitinib/placebo	PFS (cediranib + gefitinib): 3.6 m, PFS (cediranib + placebo): 2.8 m.	Cediranib and gefitinib combination improves PFS.
Badruddoja et al. [29]	2017	30	19/11	55 (18–82)	RCT (II)	MT	VEGF MGMT	Bevacizumab + TMZ	Overall response rate from diagnosis was 51 w, the PFS-6 was 52%, and median time to tumor progression was 5.5 m.	Bevacizumab + temozolomide is a salvage regimen for recurrent GBM.
Lombardi et al. [109]	2019	119	84/35	54·8 (46·8–61·3)	RCT (II)	PKP	Multitarget kinase + mTOR	regorafenib	Survival 24.8 m with regorafenib vs. 6.2 m for patients with progressive disease.	REGOMA showed survival benefits with regorafenib in recurrent GBM.
Nayak et al. [124]	2021	80	54/26	53 (42–60)	RCT (II)	IT	PD1 + VEGF	Pembrolizumab + Bevacizumab	PFS-6: 26.0% and OS 8.8 m with bevacizumab. PFS-6 was 6.7%, mOS was 10.3 m w/o bevacizumab.	Pembrolizumab +/− bevacizumab is not effective in therapy.
Weller et al. [167]	2017	745	N/A	N/A	RCT (III)	MT	EGFRvIII	TMZ +/− Rindopepimut	N/A	Rindopepimut monotherapy does not reduce GBM mortality.
Reardon et al. [136]	2020	439	235/204	55.5 (22–77)	RCT (III)	IT	PD1	Nivolumab	mOS (nivolumab): 9.8 m; bevacizumab, 10.0 m; mOS-12 42% in both groups.	Nivolumab monotherapy is as effective as bevacizumab.
D’Alessandris et al. [53]	2013	10	19/7	52.5 (29–77)	RTC	MT	VEGF + EGFRvIII	Bevacizumab + Erlotinib	mPFS 8.0 m; mOS 9.5 m.	Molecular analysis improves RR and PFS at 6 months.
Butowski et al. [38]	2010	12	8/4	11 patients > 18 and <65 y 1 patient > 65 y	RTC	PKP	Protein kinase C-beta + PI3K/Akt	Enzastaurin + TMZ	Median survival: 14.6 m, and 1/4 patients > 2 y.	Enzastaurin + RT/TMZ: Well tolerated.
Hashimoto et al. [73]	2015	7	4/3	49 (41–60)	RTC (I)	OM	WT1 (Wilms Tumor 1)	WT1 peptide vaccination + TMZ	4 patients remained in an NR status after GTR, two showed complete response.	Combined peptide vaccination with temozolomide is safe.
Desjardins et al. [55]	2011	36	29/7	52 (26–74)	RTC (I)	OM	Farnesyl transferase	SCH 66336	mOS (14.3 m); mPFS (4.5 m); PFS-6 (41.7%).	SCH 66336 dose specified for strata.
Geletneky et al. [65]	2017	18	14/4	57.8 ± 10.6	RTC (I/II)	OM	Protein NS1	Rat H-1 parvovirus (H-1PV)	PFS-6: 27%; mPFS: 111 d.	H-1PV is safe with favorable PFS.
Kanemaru et al. [90]	2019	1	1/0	N/A	Case report	PKP	BRAF + MEK	Dabrafenib + Trametinib	N/A	Dabrafenib and trametinib + radiation showed strong response in epithelioid GBM.
Anghileri et al. [26]	2021	1	0/1	N/A	Case report	IT	PD1	Nivolumab	N/A	Nivolumab is useful for GBM patients.
Johanns et al. [85]	2018	2	1/1	N/A	Case series	PKP	BRAF + MEK	Dabrafenib + Trametinib	N/A	PT1: 11mo therapy improved function, then progressed. PT2: 3 mo therapy allowed ambulation, but ended fatally.

Legend: IQR—Interquartile Range; mTOR—Mammalian Target of Rapamycin; MDM2—Mouse Double Minute 2; ALK—Anaplastic Lymphoma Kinase; CDK4/6—Cyclin-Dependent Kinase 4 and 6; SHH—Sonic Hedgehog; CSF1R—Colony-Stimulating Factor 1 Receptor; ABL—Abelson Tyrosine Kinase; cKIT—Tyrosine-protein kinase Kit; PDGFR—Platelet-Derived Growth Factor Receptor; EGFR—Epidermal Growth Factor Receptor; VEGF—Vascular Endothelial Growth Factor; VEGFR—Vascular Endothelial Growth Factor Receptor; TMZ—Temozolomide; N/A—not available or not defined.

**Table 3:** Overview of in vitro studies.

Reference	Year	Species/Culture Type	Molecular Mechanism	Molecular Target	Therapy	Success Rate/Outcome	Main Study Findings
Blank et al. [36]	2001	HCC (endothelial YPEN-1 (CRL-222), microglial cell line N9, rat GBM cell line C6)	MT	O-acetyl GD2 ganglioside	Anti-GD2 Antibody	N/A	O-acetyl GD2 ganglioside prevents glioma proliferation.
Koul et al. [95]	2005	ACC (U87, U251, LN229, SNB-19, U373, and D54 human GBM cell lines)	PKP	Integrin-linked kinase	QLT0276 In DMSO	ILK inhibition decreased the in vitro invasive capability of glioma cells, concomitant with a decrease in MMP-2 secretion.	ILK inhibition down-regulates proliferation and invasion.
Paternot et al. [128]	2009	HCC (T98G, U-87 MG, and U-138 MG)	PKP	mTOR1 + MEK1/2	Rapamycin + PD184352	Complete inhibition of DNA synthesis and pRb phosphorylation requires the combined inhibition of MEK1/2 and mTOR-raptor pathways.	Combined mTOR1 and MEK1/2 inhibition in CDK4-dysregulated tumors.
Premkumar et al. [151]	2010	HCC (U87, T98G, U373, LN229 and A172)	PKP	IGF1R + Src	NVP-AEW541 + Dasatinib	The effect on the induced formation of Bax homodimers (42 kDa), homotrimers (63 kDa), and homotetramers (84 kDa) was significantly reduced by transfection with Bcl-2 and Myr-Akt.	Dual IGF1R and Src inhibition increases apoptosis in glioma cells.
Siegelin et al. [132]	2010	HCC	PKP	BRAF	Sorafenib	N/A	Sorafenib has potent in vivo and in vitro anti-glioma activity.
Cloninger et al. [51]	2011	HCC (U87 and LN229 parental lines)	PKP	SAPK2/p38 + mTORC1	SB203580 + Rapamycin	Significant inhibition of tumor growth rate 76% (at end of dosing period) and tumor growth delay, 16.5 days.	SAPK2/p38 + mTORC1 inhibitors for synergistic response.
Liu et al. [106]	2011	HCC (U251)	PKP	bFGF	Anti-bFGF siRNA	Cytochrome C, Caspase3, and Bax were markedly higher in the Ad-bFGF-siRNA group than in the control group.	bFGF siRNA is a potential glioma treatment.
Zhang et al. [180]	2011	HCC (T98G and LN-229)	CCRM	ID2	Anti ID2 siRNA	The viability of cultured glioma cells was reduced in eEF-2 kinase knock-down when compared with control cells.	ID2 upregulation decreases glioma apoptosis; targeting increases sensitivity.
Ishiwata et al. [80]	2011	HCC and ACC/ A172	OM	hnRNP A1/B2	Β-Asarone	The growth rate and motility of Nes cells were higher than those of the mock cells	β-Asarone inhibits EMT and invasion.
Du et al. [58]	2012	HCC (BT325 and U251)	PKP	Raf/MEK/ERK signaling pathway	Sorafenib + Vitamin K (VK1)	The combination of low-concentration sorafenib (2.5 μM) and VK1 (50 μM) exhibited strong synergistic action by inhibiting protein expression of Bcl-2 and Mcl-1, leading to induction of cell apoptosis.	Sorafenib + VK1 induces apoptosis through protein regulation.
Lee et al. [101]	2012	HCC	CCRM	Wee1K	Mk-1775	The median survival time of the patients under 50 years old is 34 months, almost three times longer than the 12-month median survival time of the patients > 50.	Wee1K phosphorylation is an effective anti-tumor target.
Golubovskaya et al. [30]	2013	HCC	PKP	FAK	Y15	N/A	FAK autophosphorylation blockade with Y15 is a potential GBM therapy.
Jin et al. [83]	2013	HCC (U251, U87)	PKP	Akt + NOTCH	MRK003 + MK-2206	Combination treatment was superior to monotherapy in both U251 and U87 cells.	Akt and NOTCH inhibition decreases glioma proliferation.
Pezuk et al. [131]	2013	HCC (U251, U138, U87, T98G, U343, MO59K, LN319, SF188)	PKP	PLK1	Bi2536 + Tmz	PLK1 possible therapeutic target; BI 2536 inhibited tumor growth in vivo.	PLK1 inhibition + TMZ are effective in vitro.
Kaneta et al. [91]	2013	HCC (U1242)	CCRM	BMI-1	Ptc-209	The inhibition of NEK9 suggested as a novel anticancer therapeutic strategy.	Tumor growth is attenuated by PTC-2009; potential BMI-1 inhibitor.
Lian et al. [104]	2013	HCC	CCRM	EGFR	AZD9291	miR-23a might be employed as a novel prognostic marker and a therapeutic target for glioma.	AZD9291 is efficient in GBM preclinical models.
Mao et al. [115]	2013	HCC (U87, SF268, A172 and U118)	CCRM	MDM2/4 + α5β1/αvβ3	Compound 9	Targeting STK17A may lead to the development of new therapies for GBM and sensitize cancers to existing therapies.	Compound 9 inhibits p53, shows anti-glioma potential.
Ji et al. [82]	2013	HCC	MT	VEGFR	Axitinib	High expression levels of Nrf2 and HIF-1alpha correlated with low 1-year survival rate. median OS 13 mo.	Axitinib exhibits antiangiogenic activity and prolongs survival.
Aldea et al. [24]	2014	HCC	PKP	mTOR + RAF	Metformin + Sorafenib	Metformin + sorafenib could be combined into an efficient in vitro treatment strategy and this association is superior to either drug used alone or when compared with the use of TMZ.	Metformin + sorafenib is effective for TMZ-resistant GBM cells.
Emlet et al. [59]	2014	HCC (U87)	PKP	EGFRvIII + CD133	Egfrviii + CD133 AB	The specific lysis of the EGFRvIII+/CD133+ population significantly reduces the implantation of primary GBM tumors in mice and prolongs survival.	EGFRvIII + CD133 BsAb target cancer stem cells.
Hong et al. [77]	2014	HCC	PKP	Aurora-A kinase	Alisertib	CE7 epitope was highly detected in GBM and it represents a potential therapeutic target.	Inhibiting Aurora-A kinase enhances radiation effects.
Jung et al. [89]	2014	HCC	PKP	FOXO3A	Z-Ajoene	Z-ajoene is a potential candidate for the treatment of GBM.	Z-ajoene targets glioma CSCs via FOXO3A pathway.
Liu et al. [105]	2014	HCC (CE7R+ T cells)	PKP	EGFR and PI3K/Akt	G19	G19 inhibited cell proliferation of U-87 MG human glioma cells in vitro and in vivo	G19 targets EGFR and PI3K/Akt, inducing redox stress.
Liu et al. [108]	2014	HCC (T98G, A172, and U87)	PKP	AMPK	Compound C	Compound C at 10 μM inhibited proliferation and glioma formation of human U87MG glioma cells in vivo.	Compound C is a potent anti-glioma agent.
Camorani et al. [40]	2015	HCC (U87MG)	PKP	EGFRvIII	CL4 Aptamer + EGFR Tkis	Combined treatment with CL4 and Gint4.T aptamers led to a consistently higher inhibition of cell growth.	CL4 and gefitinib cooperate with anti-PDGFRβ aptamer.
Ma et al. [114]	2015	HCC (U251 and U87)	PKP	STAT3	Tetrandrine	Higher expressions of STAT3 in patients with glioma received lower survival rates.	Tetrandrine inhibits glioma growth without affecting embryos.
Wichmann et al. [168]	2015	HCC (U251 and LN-229)	PKP	EGFR and HER2	siRNA + Cetuximab + Trastuzumab	Knock-down of HER2 reduces clonogenic survival in both GBM cell lines.	EGFR and HER2 siRNA reduce GBM growth rate.
Zhao et al. [181]	2015	HCC (U87)	CCRM	CDK + Aurora (dual inhibitor)	Jnj-7706621	Id2 is a good molecular target for GBM gene therapy.	JNJ-7706621 shows potential for GBM treatment.
Xu et al. [172]	2015	HCC (U87MG)	IT	CXCR4	POL5551 + MCR89	Icaritin is a promising anti-cancer agent in the treatment of GBM.	Higher POL5551 concentrations improve survival, especially with VEGF antagonism.
Junca et al. [88]	2017	HCC	PKP	ALK, ROS1, MET	Crizotinib	Overexpression was associated with poor prognosis with a survival of 11.7 months against 14.3 months for patients whose tumors did not express or had low expression of MET.	MET and ALK overexpression in glioma; crizotinib potential.
Thanasupawat et al. [154]	2017	HCC (U87MG)	PKP	FGFR	Dovitinib	N/A	Alternation of dovitinib and TMZ reduces GBM viability.
Caruana et al. [41]	2017	HCC (T98G)	OM	APLNR	MM54 Or MM193 (APLNR Antagonists)	N/A	APLNR inhibition significantly reduces tumor growth.
Barbarisi et al. [32]	2018	HCC	PKP	CD44	Quercetin + TMZ	N/A	CD44-targeted nanocarriers deliver quercetin to GBM.
Merlino et al. [119]	2018	HCC (U87MG)	CCRM	CDK 4/6	PD-0332991	N/A	PD-0332991 inhibits glioma growth, increases survival.
Franco et al. [63]	2018	HCC (U87MG)	MT	LTβR	Light-VTP	N/A	LIGHT-VTP prevents angiogenesis and promotes immune infiltration.
Pall et al. [127]	2019	HCC (hBMVECs, U251n and U87, RAW264.7)	MT	HIF2α	PT2385	N/A	HIF2α is a reasonable therapeutic target; PT2385 is effective.
Xiong et al. [171]	2019	HCC (MCF7, HL60, MCF7)	MT	STING	ASA404	N/A	ASA404 efficacy varies by administration method.
Peng et al. [129]	2019	HCC (U-373MG Uppsala, U-87MG Uppsala, U251 and T98G)	OM	EFTUD1	EFTUD1 shRNA	N/A	EFTUD1 overexpression is associated with glioma.
Ariey-Bonnet et al. [28]	2020	HCC (U87, U87vIII, T98G, and U251)	PKP	MAPK14	BMZ	N/A	BMZ inhibits MAPK14, with anticancer properties.
Bagca et al. [30]	2020	HCC (T98G)	PKP	ALK	AZD3463 + TMZ	N/A	Combo with AZD3463 may enhance TMZ in GBM.
Bychkov et al. [39]	2020	HCC (U251 MG and A172)	CCRM	S100A9 (one of the heterodimers for calprotectin)	shRNA	N/A	S100A9 knockdown demonstrates anticancer potential.
Cheng et al. [47]	2022	HCC (LN-229, T98, A172, and human astrocyte)	PKP	CTSC	Piperlongumine + Scopoletin	N/A	CTSC is a MAPK biomarker; piperlongumine and scopoletin inhibit growth.

Legend: HCC—Human Cell Culture; ACC—Animal Cell Culture; CCRM—Cell Cycle Regulation Mechanism; PKP—Protein Kinase Pathway; MT—Molecular Targeting; OM—Oncogene Mutation; IT—Immunotherapy; LTβR—Lymphotoxin Beta Receptor; ALK—Anaplastic Lymphoma Kinase; MAPK—Mitogen-Activated Protein Kinase; CTSC—Cathepsin C; CDK—Cyclin-Dependent Kinase; FGFR—Fibroblast Growth Factor Receptor; S100A9—S100 Calcium-Binding Protein A9; CXCR4—C-X-C Chemokine Receptor Type 4; AMPK—AMP-Activated Protein Kinase; MDM2/4—Mouse Double Minute 2/4; SAPK2/p38—Stress-Activated Protein Kinase 2/p38; hnRNP—Heterogeneous Nuclear Ribonucleoprotein; VEGFR—Vascular Endothelial Growth Factor Receptor; FOXO3A—Forkhead Box O3A; CD44—Cluster of Differentiation 44; CD133—Cluster of Differentiation 133; EGFR—Epidermal Growth Factor Receptor; EGFRvIII—Epidermal Growth Factor Receptor Variant III; IGF1R—Insulin-Like Growth Factor 1 Receptor; Src—Proto-Oncogene Tyrosine-Protein Kinase Src; RAF—Rapidly Accelerated Fibrosarcoma; MEK—Mitogen-Activated Protein Kinase; mTOR—Mammalian Target of Rapamycin; PD—Phosphoinositide; Akt—Protein Kinase B; PI3K—Phosphoinositide 3-Kinase; MRP—Multidrug Resistance-Associated Protein; PKB—Protein Kinase B; MEK1/2—Mitogen-Activated Protein Kinase 1 and 2; ERK—Extracellular Signal-Regulated Kinase; EMT—Epithelial-Mesenchymal Transition; Nrf2—Nuclear Factor Erythroid 2-Related Factor 2; HIF-1alpha—Hypoxia-Inducible Factor 1 Alpha; ILK—Integrin-Linked Kinase; FAK—Focal Adhesion Kinase; MET—Mesenchymal-Epithelial Transition Factor; STK17A—Serine/Threonine Kinase 17A; BMI-1—B-Lymphoma Mo-MLV Insertion Region 1; APLNR—Apelin Receptor; JNJ-7706621—a dual CDK and Aurora Kinase inhibitor; MK-2206—an Akt inhibitor; Light-VTP—Light-Photochemical Internalization; STAT3—Signal Transducer and Activator of Transcription 3; PD-0332991—Palbociclib; STING—Stimulator of Interferon Genes; ASA404—Vadimezan; EFTUD1—Elongation Factor Tu GTP-Binding Domain-Containing 1; N/A—not available or not defined.

**Table 4:** Overview of in vivo studies.

Reference	Year	Species/Culture Type	Molecular Mechanism	Molecular Target	Therapy	Success Rate/Outcome	Main Study Findings
Takano et al. [152]	2003	A (mice)	MT	CTL1 (choline transporter-like protein 1)	AMB4269951	MST: 52.8 ± 5.5 days (ACNU + VEGF therapy significantly mouse survival.	Amb4269951 has significant antitumor effects in glioma.
Saito et al. [139]	2004	A (rats)	OM	α7 nAChR	Rslurp-1	Rats who received combination therapy survived more than 80 days and revealed fibrous scar tissue at necropsy.	rSLURP-1 demonstrates antitumor activity.
SathornSumetee et al. [143]	2006	A (mice)	PKP	BRAF, CRAF, VEGFR	AA1881	Median life spans of 12 days for control mice and 44 days for AAL881-treated animals.	AAL881 inhibits glioma growth, well tolerated.
Yang et al. [175]	2006	A (rats)	CCRM	EF2-kinase	EF2-siRNA	MST (bioconjugate in combination with BPA) = 85.5 days compared with 70.4 days for those that received it alone, 40.1 days for BPA alone, and 30.3 days for irradiated controls.	EF2 regulates cell migration; knockdown inhibits these properties.
Yang et al. [176]	2008	A (rats)	PKP	EGFR	Boronated EGFR MAB + Cetuximab	The MST of animals that received both boronated mAbs was 55 days compared with MSTs of 36 and 38 days for animals that received either one or the other boronated mAb.	Both EGFR and EGFRvIII tumors must be targeted for glioma.
Feng et al. [61]	2010	A (Rats)	PKP	PI3K/Akt; JNK; ERK	Tamoxifen	Treatment with TAM at 20 μM caused about half of the C6 glioma cells to die after 24 h.	TAM-induced apoptosis reveals potential targets.
Koul et al. [96]	2010	A (mice)	PKP	PI3K/Akt	Px-866	The median survival time for controls was 32 days; treated with PX-866 was significantly longer at 39 days.	PX-866 inhibits growth, induces G1 arrest in mice.
Colen et al. [52]	2011	HCC (U87-MG and U251-MG)	OM	MALAT1	Nanocomplex Targeting MALAT1 + TMZ	A 50% survival rate was observed with the nude rat model with no tumor recurrence after treatment.	Combined TMZ with MALAT1 silencing offers a survival benefit.
Joshi et al. [86]	2012	HCC (serum-cultured and oncosphere lines)	PKP	Multitarget kinases	Gefitinib + Erlotinib + Sunitinib	Use of PAC-1 with TMZ substantially improved the median survival to 205 days compared to vehicle, compared to TMZ alone.	Sunitinib combinations are effective in vitro, not in vivo.
Li et al. [103]	2012	A (mice)	CCRM	miR-23a (APAF1)	Anti-mir-23a	The MST of intracranial U87 glioblastoma-bearing nude mice treated with RGD-liposomal pDP (29 days) was significantly longer than that of mice treated with blank RGD-liposome (23 days) (*p* < 0.001).	miR-23a upregulated in gliomas; knockdown reduces survivability.
Arcella et al. [27]	2013	A (mice)	PKP	mTOR	Rapamycin	Rapamycin-treated mice survive almost double that observed in vehicle-treated mice.	Rapamycin is a potent mTOR inhibitor for GBM.
Grossman et al. [69]	2013	A (rats)	MT	TRPV4	Cannabidiol (CBD)	TMZ intratumoral concentrations do not decline in the setting of the oral tyrosine kinase inhibitor cediranib.	CBD induces lethal mitophagy; TRPV4 is a target.
Chen et al. [46]	2013	A (rats)	OM	Nestin	Anti-Nestin IGG	TMP shown to be a potential therapeutic candidate for the treatment of resistant malignant gliomas.	Nestin downregulation is associated with reduced glioma proliferation and migration.
Wang et al. [162]	2014	H	PKP	RAS	Mir-143	ER 51.6% ER 63.6% ER 32.0%	miR-143 downregulated in glioma, inactivates RAS.
Shingu et al. [149]	2015	A (mice)	PKP	MEK, EGFR, PI3K	Various Small Molecule Inhibitors	Combination of erlotinib and sorafenib tended to improve survival of nude mice bearing GSC11 brain tumors.	Most synergistic drug combinations affect RTKs and MEK/ERK or PI3K.
Yao et al. [177]	2015	A (mice)	PKP	EGFR and BRAF	BRAF(V600E) Inhibitor PLX4720	BRAFV600E + EGFR inhibitors showed dramatic reduction in tumor growth and extended survival compared to vehicle or single-drug-treated counterparts.	Inhibiting EGFR and BRAF(V600E) reduces proliferation.
Venere et al. [157]	2015	A (mice)	OM	IDH1R132H	Wm17	median survival of 36 days versus 24 days for the DMSO vehicle cohort.	WM17 is a mutant IDH1 inhibitor.
Balkhi et al. [113]	2016	A (rat)	PKP	Multitarget kinases	Caffeic Acid Phenethyl Ester (CAPE) + Dasatinib	N/A	Combo therapy inhibits migration and invasiveness, and reduces survival.
He et al. [76]	2016	A (mice)	PKP	MEK2	MEK2 Antibody	Si-MEK2-infected U87 cell glioma burden mice had longer survival times (48 d) compared with Si-ctl-infected glioma burden mice (30.4).	MEK2 antagonists sensitize TMZ treatment in GBM.
Ju et al. [87]	2016	A (mice)	PKP	COX-2	Celecoxib	Physiological saline survival (days): range 13–25, Targeting epirubicin + celecoxib liposomes survival (days): 15–36.	Targeting epirubicin plus celecoxib liposomes effective in glioma.
Zhang et al. [178]	2016	A (mice)	PKP	HER2	HER2-specific NK cells	Median survival of 200.5 days upon treatment with NK-92/5.28.z vs 73 days upon treatment with parental NK-92 cells.	Modified HER2-specific NK cells effective against GBM.
Grinshtein et al. [68]	2016	A (mice)	CCRM	BAG3	BAG3 siRNA	N/A	BAG3 is highly expressed in gliomas; a therapeutic target.
Lescarbeau et al. [102]	2016	A (mice)	CCRM	p53/MDM2	D-PMNIbeta	Mean survival time after treatment initiation was 22 days with MK-1775 treatment and only 13 days with control.	D-PMIBeta an effective p53 inhibitor.
Tchoghandjian et al. [153]	2016	A (mice)	CCRM	EGFR	Afatinib + TMZ	Treatment significantly increased mouse survival in a dose-dependent manner.	Afatinib + TMZ significantly delays progression.
Fleurence et al. [62]	2016	A (mice)	MT	Pan-VEGF	Cediranib + TMZ	N/A	Intratumoral TMZ concentrations are slightly increased.
Farrell et al. [60]	2017	A (mice)	PKP	MET	WO2010/019899A1 + PF04217903 + Crizotinib	N/A	Dual targeting of HGF and MET could be effective.
Yan et al. [174]	2017	A (mice)	PKP	CSF-1R + cKIT + RTKs	PLX3397 + Vatalanib + Dovitinib	N/A	PLX3397 is effective, improves TKI efficacy.
Joshi et al. [86]	2017	A (mice)	CCRM	Phospholipase C	D609	Rats treated with PAC-1 showed significantly improved survival (59 d) was 350% longer than for the untreated control rats (17 d), and 40% of the animals survived until the end of the experiment (day 200).	Chronic D609 treatment leads to decreased Olig2 biomarker levels.
Abdul Rahim et al. [23]	2017	A (mice)	MT	Phosphatidylserine	SAPC-DOPS	ATG9A knockdown led to a significant increase in mouse survival (+12–18%, Chloroquine treatment (20 mg kg−1) significantly prolonged survival of P3 mice (+18.4%).	SAPc-DOPS targets GBM effectively.
Angara et al. [25]	2017	A (rat)	MT	Endothelial pigpen protein	Aptamer III.1	N/A	Aptamer III.1 is a potential GBM treatment.
Bäehr et al. [31]	2017	A (mice)	IT	ATX + LPA receptors	siRNA	Median survival was 36 days for PBS and 40 days for ASA404.	ATX and LPA receptor downregulation enhances radio-sensitivity.
Harford-Wright et al. [72]	2017	A (mice)	OM	IDH1R132H	AGI-5198 (In Combo with HDACi)	N/A	AGI-5198 attenuates HDACi resistance.
Tu et al. [156]	2017	A (mice)	OM	14-3-2003	siRNA	N/A	14-3-3 downregulation decreases glioma survival.
Ciesielski et al. [49]	2018	A (mice)	PKP	Src-kinase + tubulin polymerization inhibitory activity	Kx2-361	N/A	Active against GL261 gliomas in mice.
Kong et al. [94]	2018	A (mice)	CCRM	OPN	shRNA	N/A	U87-MG sphere cells’ tumorigenic potential abrogated upon OPN silencing.
He et al. [75]	2018	A (mice)	MT	VEGF + Src Family kinases	Bevacizumab + Dasatinib	N/A	Dasatinib may block bevacizumab-induced invasion.
Nandhu et al. [123]	2018	A (mice)	MT	NHE9	Gold NEPTT	Intravenously injected mAb428.2 reduced tumor volume and significantly improved survival in all the fibulin-3-expressing models, extending median survival by 28% (GBM09) to 64% (GBM34) in the GBM xenografts. However, mAb428.2 did not prolong the survival of mice carrying fibulin-3-negative COLO201 tumor cells.	Gold nanoparticle-enabled photothermal therapy (NEPTT) kills tumor cells.
Kim et al. [93]	2018	A (mice)	OM	LPAR1/3	KI16425	Survival was extended by scL-siMAL + TMZ (50% of the mice were surviving at day 38).	LPA signaling knockdown reduces tumor growth.
Loskutov et al. [110]	2018	A (mice)	OM	PRC2 + BET bromodomain proteins	JQ1 + I-BET	N/A	H3K27M mutation effects are reduced by inhibiting PRC2 and BET proteins.
Chen et al. [44]	2019	A (mice)	PKP	CD163 pathway (CK2, kinase)	TBB	N/A	TBB inhibits CK2, crucial for tumor growth.
Chen et al. [42]	2019	A (mice)	CCRM	HDAC/EZH2	Compound 26/UNC1999	IGFBP3 siRNA-treated mice showed better OS compared with siCtrl-treated mice, and the median survival of siCtrl-, siIBP3-1-, and siIBP3-2-treated mice was 25, 32, and 35 d.	HDAC and EZH2 inhibition shows synergistic effects.
Liu et al. [107]	2019	A (mice)	CCRM	STK17A	Anti-STK17A shRNA	N/A	STK17A indicates worse prognosis; knockdown reduces survivability.
Vengoji et al. [158]	2019	A (mice)	CCRM	Survivin	Survivin-siRNA/Transferrin Receptor Conjugate	N/A	Conjugate decreases survivin expression, increases survival.
Wang et al. [163]	2019	A (mice)	CCRM	Carbamoyl-phosphate synthetase (CAD)	Teriflunomide	N/A	Targeting pyrimidine synthesis may improve outcomes.
Xu et al. [173]	2019	A (mice)	MT	CD73	Anti-CD73	N/A	Combination therapy targets CD73; anti-CD73 testing suggested.
Luwor et al. [112]	2019	A (mice)	OM	eIF-5A, DHS, DOHH (both eIF-5A activators)	Gc7	N/A	eIF5-A is a potential therapeutic target.
Selvasaravanan et al. [148]	2020	A (mice)	PKP	MEK or PI3K	Trametinib + Pictilisib	N/A	MEK inhibition is not superior to PI3K inhibition.
Punganuru et al. [134]	2020	A (mice)	CCRM	HSP90	BIIB021 + 17-AAG (HSP90 Inhibitor) + BRAFi +/Or MEKi	N/A	HSP90 inhibitor overcomes limitations of BRAFV600E therapy.
Renfrow et al. [137]	2020	A (mice)	MT	VEGF	Anti-VEGF AB + Nimustine	PT2385 single-agent treatment did improve mOS compared to placebo; no difference in animal survival was seen in combination treatment with radiation (RTtemozolomide TMZPT2385).	Combining antiangiogenic therapy with chemotherapy is promising.
Watanabe et al. [166]	2020	A (mice)	MT	Calmodulin, EGFR, aromatase	W-13 + Gefitinib + Exemestane	N/A	Identified miRNA-based chemicals for therapy.
Goswami et al. [67]	2020	A (mice)	IT	EMMPRIN	Icaritin	Improvement in survival was noted in WT and CD73−/− mice treated with anti-PD-1 + anti-CTLA-4 compared to untreated controls. Following treatment of anti-PD-1 + anti-CTLA-4, CD73−/− mice had improved survival as compared to WT GBM tumor-bearing mice.	Icaritin targets EMMPRIN, inhibiting GBM cell invasion and EMT.
Shulepko et al. [150]	2020	H	OM	KIF11	Ipinesib	N/A	KIF11 inhibition halts tumor growth.
Kawauchi et al. [92]	2021	A (mice)	PKP	ALK	Alectinib + Ceritinib	Ceritinib or alectinib significantly prolonged the survival of mice harboring intracerebral U87MG or GSC23 xenografts.	Second-gen ALK inhibitors are potent against GBM.
Maxwell et al. [118]	2021	A (mice)	PKP	mTOR1/2 + MEK	TAK228 + Trametinib	N/A	mTOR1/2 and MEK inhibitors induce proteomic changes.
Genoud et al. [66]	2021	A (mice)	CCRM	PAK5	PAK5 shRNA	Significant increase in survival with BAL101553 + aCD40 (49 d), compared with BAL101553 monotherapy (42).	PAK5 overexpressed in glioma; its inhibition is promising.
Huang et al. [78]	2021	A (mice)	MT	Growth-Hormone Releasing Hormone	MIA-604 + MIA-690	N/A	GHRH antagonists augment standard treatments.
Chen et al. [45]	2021	A (mice)	OM	EEF1A1 + RPL11	Puromycin + Doxorubicin + Daunorubicin + Mitoxantrone	N/A	Database analysis identifies target genes and potential drugs for glioma treatment.
Saunders et al. [144]	2021	A (mice)	OM	Smoothened	Gdc-0449	NSC682769 treated GFAP-EGFRvIII × GFAP-Cre+/Rictor mice had a marked increase in OS with more than 75% of mice surviving at 20 weeks at 20 mg/kg and 60% of mice surviving to 20 weeks receiving the lower 5 mg/kg regimen.	Smoothened is a prognostic biomarker.
von Spreckelsen et al. [159]	2021	A (mice)	OM	FTO	SPI1 Inhibitor DB2313	N/A	FTO is a novel prognostic indicator.
Xia et al. [170]	2022	A (mice)	MT	ITGA9	miR-148a	U251 + U87 cell survival overexpressing Nrf2 remarkably increased 24, 48, and 72 h after treatment with apatinib, in comparison with cells transfected with the empty vector.	miR-148a suppresses GBM malignancy via ITGA9 targeting.
Wang et al. [164]	2022	A (mice)	CCRM	BCL6	RI-BPi	The combination of lapatinib and teriflunomide yielded the greatest efficacy in tumor control and OS.	BCL6 overexpression in glioma worsens prognosis; RI-BPI reduces tumor growth.
Joel et al. [84]	2015	N/A	PKP	PBK/TOPK	Hi-Topk-032	PBK may serve as a potential therapeutic target in GBM tumors.	HITOPK-032 diminishes tumor growth.

Legend: A—Animal; CCRM—Cell Cycle Regulation Mechanism; MT—Molecular Targeting; OM—Oncogene Mutation; PKP—Protein Kinase Pathway; EGFR—Epidermal Growth Factor Receptor; VEGFR—Vascular Endothelial Growth Factor Receptor; PI3K—Phosphoinositide 3-Kinase; TMZ—Temozolomide; DNA—Deoxyribonucleic Acid; RNA—Ribonucleic Acid; PCR—Polymerase Chain Reaction; mRNA—Messenger Ribonucleic Acid; rRNA—Ribosomal Ribonucleic Acid; tRNA—Transfer Ribonucleic Acid; ICU—Intensive Care Unit; ER—Emergency Room; AIDS—Acquired Immunodeficiency Syndrome; CD4—Cluster of Differentiation 4; N/A—not available or not defined.

**Table 5:** Overview of combined (in vivo and in vitro) studies.

Reference	Year	Species/Culture Type	Molecular Mechanism	Molecular Target	Therapy	Success Rate/Outcome	Main Study Findings
Kuan et al. [97]	2010	HCC (D54 MG, D247 MG, D392 MG, and D245 MG, T98G and U251 MG	MT	TfR (transferrin receptor)	T12 + B6 + T7 (Tfr-Targeting Peptides)	High levels of RNA remain a significant predictor of survival.	T7-modified liposomes penetrate the BBB effectively.
Guo et al. [71]	2011	HCC (U87MG)	CCRM	CDK 4/6 + PDGFRα	Lenvatinib + Crenolanib + Abemaciclib + Palbociclib	Compared to physiological saline, DOX-LP+ TRAIL-LP, DOX-LP, and DOX significantly prolonged the survival time (48,49,36 days).	PDGFRα and CDK4/6 signaling blockade for a splice variant.
Wang et al. [161]	2011	HCC (U87)	CCRM	EZH2	EZH2si-DMC	Decrease in proliferation of cancer cells, reduction in cancer cell survival in vitro, and a reduction in tumor volume in nude mice.	DMC nanoparticle-mediated EZH2-siRNA decreases tumor size.
Schleicher et al. [146]	2011	HCC (Endothelial cells, HUVEC) and ACC (mice) (bEND.3)	IT	FPR	F2 Procyanidins	Mice treated with BrP-LPA and irradiation showed a tumor growth delay of 6.8 days compared to mice treated with irradiation alone.	F2 procyanidins downregulate FPR, exerting a cytotoxic effect.
Benezra et al. [34]	2012	HCC	PKP	Multitarget kinases	Dasatinib	Mice gavaged with saline vehicle survived 14 to 17 days posttreatment, whereas dasatinib-gavaged mice survived 18 to 30 days.	Dasatinib boosts survival in mouse GBM.
Matsuda et al. [117]	2012	HCC (TGS01, GS-Y01)	PKP	JNK	Sp600125	All mice survived beyond 12 months after treatment, with no significant differences found in general health status as assessed by body weight and survival, and in cognitive function.	JNK is a target for stem-like potential in GBM.
Salphati et al. [140]	2012	HCC (MDR1-MDCKI, Bcrp1-MDCKII, Bcrp-MDCKII, Mdr1a-LLC-PK	PKP	PI3K	Gne-317	In the GBM10 model, mice that were treated with GNE-317 experienced a marked survival benefit.	GNE-317 is a PI3K inhibitor for GBM.
See et al. [147]	2012	HCC (TCC; LN229, A172, T98G, MO59J, LN18, U87, U138) or the UCSF BTRC Tissue Core (U251, U373, SF188, U343, SF126, SF210, SF268, SF295, SF539)	PKP	MEK + PI3K/mTOR	Vemurafenib + PI103	PD0325901 suppressed the growth of LN229 tumors and increased the survival of LN229-bearing animals, but had no significant effect on the intracranial growth of U251 cells or the survival of U251 tumor-bearing mice.	MEK inhibitor-resistant GBM lines respond to dual therapy.
Miyazaki et al. [121]	2012	HCC (GBM146, 157, 205, 206, 218, 1600, 2313, and 13, f16w and 1105A)	OM	TRAILR	Recombinant TRAIL + TMZ	N/A	TMZ + TRAIL synergistically improve survival in tumor-bearing rats.
Preukschas et al. [133]	2012	H/HCC (G55T2, U87-MG)	OM	YAP1	Nsc682769	Glioma patients with a high expression of eIF-5A have a lower probability of survival, compared to patients with an intermediate expression.	NSC682769 is a YAP1 inhibitor, decreasing glioma growth.
Dominguez et al. [57]	2013	HCC (U87, U251)	PKP	DGK-α	R59022 + R59949 + siRNA	N/A	DGK-α is a potential glioma target linked to pathways.
Peng et al. [130]	2013	HCC (U87 and CHG-5)	PKP	RACK1-PKC	siRNA	N/A	RACK1 is a glioma development target via SRC/Akt activity.
Chen et al. [43]	2013	HCC (U87)	MT	TFAM	Melatonin + TMZ	N/A	Melatonin enhances TMZ effects via TFAM inhibition.
Huveldt et al. [79]	2013	HCC	MT	Nrf2	siRNA	Dasatinib effectively blocked the increased invasion induced by bevacizumab, thus its combination is recommended to be used in clinical settings.	Nrf2 promotes glioma proliferation; siRNA is a potential drug.
Jaszberenyi et al. [81]	2013	HCC (U-87)	MT	MRP3	Anti-MRP Antibody	GHRH antagonists can increase the direct inhibitory effect of traditional chemotherapeutic drugs.	MRP3 is overexpressed in gliomas; specific antibodies decrease growth.
Luchman et al. [111]	2011	HCC (BT142)	PKP	mTOR1/2	AZD8055	N/A	Dual mTOR1/2 inhibition + TMZ for resistant GBM.
Qin et al. [135]	2014	A (mice and rats) + HCC	PKP	EMP2	Anti-EMP2 antibodies/Anti-EMP2 Igg1	Abemaciclib + TMZ increased survival by 31–37,5 days.	EMP2 promotes migration/invasion via protein kinases.
Signore et al. [151]	2014	HCC (U87MG)	PKP	PDK1 + CHK1	UCN-01	Combined inhibition of PDK1 and CHK1 represents a potentially effective therapeutic approach to growth reduction of human GBM.	UCN-01 downregulates PDK1 and CHK1, killing tumor cells.
Blanco et al. [35]	2014	HCC (U87ΔEGFR-Luc, U87-MG)	MT	NRP-1	NRP-1 Mab	SapC-DOPS nanovesicles target tumor cells and exert antitumor actions both in vitro and in vivo	NRP-1Mab inhibits glioma growth and invasion.
Barone et al. [33]	2014	HCC (U87)	OM	Lactate (monocarboxylate) transporters	ACCA	CXCR4 antagonist + POL5551 + mcr84 can increase median OS in GBM xenografts compared to treatment with either drug as monotherapy.	ACCA inhibits lactate transport, a potential brain tumor target.
Saito et al. [138]	2014	HCC (U87MG and T98G)	OM	A1CF + FAM224A	shRNA	Combination therapy consisting of EFTUD1 downregulation with an autophagy blocker enhanced the antitumor effect.	A1CF/FAM224A/miR-590-3p/ZNF143 loop regulates tumor progression.
Di Stefano et al. [56]	2015	HCC	PKP	FGFR kinase	JNJ-42756493	Targeted inhibition of FGFR-TK with JNJ-42756493 may provide clinical benefits for patients with recurrent glioma.	JNJ-42756493 inhibits growth and regression in GBM.
Zhang et al. [179]	2015	HCC (U87)	PKP	mGluR1	siRNA, Selective Antagonists Riluzole + BAY36-7620	mGluR1 is a potential therapeutic target for the treatment of human gliomas.	mGluR1 inhibition demonstrated antitumor activity.
Ge et al. [64]	2015	HCC (U87)	CCRM	Tumor checkpoint controller targeting microtubules	BAL101553	anti-miR-27a could inhibit the growth of GBM and has potential for clinical application.	BAL101553 is a promising GBM agent.
Gu et al. [70]	2015	HCC (U87, SHG-44, CHG-5, and U251)	CCRM	DR4/5	TRAIL + Doxorubicin	Inhibition of PAK5 by lentivirus-mediated RNAi suppressed glioma development.	TRAIL-LP and DOX-LP are stronger against GBM in vivo.
Lamour et al. [98]	2015	HCC (U87-MG and U251-MG)	CCRM	PLK1	Bi2536	N/A	PLK1 is critical to glioma cell survival.
Niu et al. [125]	2015	HCC (U87, A172, SHG44, and U251)	CCRM	XIAP + BCL-2	RIST + ARIST	Plumbagin inhibited glioma cell proliferation and promoted apoptosis in a nude mouse model.	RIST and aRIST prolong survival, reduce tumor burden.
Nonnenmacher et al. [126]	2015	HCC (A172, D54, U118, U138, T98G, U87-MG)	CCRM	MGMT	PRIMA-1MET	RIST therapy can be considered a promising treatment strategy for GBM.	PRIMA-1MET targets p53, an effective therapy.
Sanzey et al. [142]	2015	HCC (NCH421k, NCH660h, NCH465, NCH601 and NCH644	OM	DLL3	Rova-T	Glycolysis is a promising target for GBM therapy.	DLL3 is selectively expressed in glioma; targetable with Rova-T.
Tsigelny et al. [155]	2017	HCC and ACC (mice GBM4, GBM8, U87, and NHA)	PKP	OLIG2	SKOG102	N/A	SKOG102 inhibits glioma growth via OLIG2 downregulation.
Mojarad-Jabali et al. [122]	2022	HCC (T7, B6, and T12 peptides)	MT	Fibulin-3	Mab428.2	N/A	mAb428.2 inhibits fibulin-3, reduces tumor growth, and extends survival.
Michaud et al. [120]	2010	HCC (U87MG, U138MG, M059J, Hs683, H4, A172, LN18, LN229, CCF-STTG1, T98G, DBTRG-05MG, 8MGBA, 42MGBA, DKMG, GAMG, GMS10, LN405, SNB19, AM38, NMC-G1, and KG-1-C)	CCRM	FOXM1	Plumbagin	The antitumor activity of PD0332991, when used with radiation either concurrently or sequentially, is superior to monotherapy.	Plumbagin inhibits glioma proliferation, induces apoptosis.

Legend: HCC—Human Cell Culture; PKP—Protein Kinase Pathway; OM—Oncogenic Mutations; CCRM—Cell Cycle Regulation Mechanism; ACC—Animal Cell Culture; AMP—AMP-Activated Protein Kinase; WNT—Wnt Signaling; NK—Natural Killer; CIC—Cancer-Initiating Cell; NKCC—Norepinephrine Kinase Complex Cell; ECM—Extracellular Matrix; BBB—Blood-Brain Barrier; MGMT—O6-Methylguanine-DNA Methyltransferase; EMT—Epithelial-Mesenchymal Transition; N/A—not available or not defined.

## Data Availability

Data are contained within the article.

## Appendix A

**Search Strategy**	
Search	(Glioblastoma multiforme OR GBM) AND (Molecular targeted therapy)
Filter	from 2000 to 2022
Search details	((“glioblastoma”[MeSH Terms] OR “glioblastoma”[All Fields] OR (“glioblastoma”[All Fields] AND “multiforme”[All Fields]) OR “glioblastoma multiforme”[All Fields] OR “GBM”[All Fields]) AND (“molecular targeted therapy”[MeSH Terms] OR (“molecular”[All Fields] AND “targeted”[All Fields] AND “therapy”[All Fields]) OR “molecular targeted therapy”[All Fields] OR (“protein kinase inhibitors”[Pharmacological Action] OR “protein kinase inhibitors”[MeSH Terms] OR (“protein”[All Fields] AND “kinase”[All Fields] AND “inhibitors”[All Fields]) OR “protein kinase inhibitors”[All Fields]) OR (“immunotherapy”[MeSH Terms] OR “immunotherapy”[All Fields] OR “immunotherapies”[All Fields] OR “immunotherapy s”[All Fields]) OR (“apoptosis”[MeSH Terms] OR “apoptosis”[All Fields]))) AND (2000:2022[pdat])
